# Social prescribing for refugee populations: a protocol for a rapid realist review of international evidence

**DOI:** 10.3389/fpubh.2026.1754718

**Published:** 2026-02-16

**Authors:** Victoria Touzel, Anna-Lena Esser, Kerryn Husk, Doreen Reifegerste

**Affiliations:** 1 AG4 Prevention and health promotion, Bielefeld University, Bielefeld, Germany; 2 Faculty of Health, University of Plymouth, Plymouth, United Kingdom

**Keywords:** asylum seeker, realist review, refugee, social capital, social prescribing

## Abstract

**Background:**

Social prescribing addresses complex individual health and social needs through person-centered referral from healthcare into community settings, supporting wellbeing and integrated care. While evidence for majority populations continues to expand, no review has yet synthesized evidence specifically for refugee populations, despite this group facing profound social and systemic barriers. As such, this protocol defines the research objectives for a rapid realist review that will identify what approaches to social interventions work for refugee populations, for whom they are effective, and under which circumstances.

**Methods:**

A rapid realist review will be conducted to identify relevant evidence from both social prescribing interventions and social capital-based interventions (where comparably operationalized), published 2014–2024. The date range reflects recent expansion of both social prescribing interest and forced migration research, with earlier intervention being less commonly documented. Six databases will be searched (PubMed, Web of Science, Embase, CINAHL, SCOPUS, PsycInfo), complemented by extensive supplementary search strategies. Two independent reviewers will screen and extract data using piloted criteria.

**Synthesis:**

Realist analysis will map included evidence to identify intervention concepts, methodologies, populations, settings, delivery structures, and evaluation measures. This mapping will be used to identify and group families of interventions that function comparably and to develop program theories using if-then logic (statements theorizing how social prescribing programs may be effective using a context-mechanism-outcome configuration). Synthesis results will include multiple products to transparently evidence the working process of the review and to illustrate the final results.

**Dissemination:**

Findings will be published in a peer-reviewed, open-access journal, with a briefing paper distributed to relevant research networks.

**Systematic review registration:**

https://doi.org/10.17605/OSF.IO/PTKDX.

## Introduction

### Background

Social prescribing addresses complex individual health and social needs in a person-centered framework, as an umbrella term for the diverse practices of referring from (often primary health) care settings into non-clinical settings. It is a multi-level approach, whereby individuals are supported to better understand and manage their own health and wellbeing, while at societal level the practice can facilitate greater collaboration and promote integrated care (1). On an individual level, social prescribing aims to create new connections to community resources for individuals who are experiencing greater levels of social need (2, 3). These interactions between the individual and the community social groups or services aim to support greater well-being and build capacity for agency, health literacy, and self-management outside of clinical settings (1, 2, 4). The greatest amount of meta-analysis evidence available focuses on adult and older adults majority populations with no review yet synthesizing evidence for social prescribing evidence for refugees.

### Barriers faced by refugee populations

Refugee populations often face greater social and systemic barriers than most majority populations (5–8). Specifically, they experience compounding barriers that distinguish their needs from majority population, including language barriers, cultural differences, and structurally dictated restrictions to agency (e.g., mobility, financial, digital access) (9–11). The impacts of these barriers may interact syndemically with other eco-social factors such as individual mental health needs, lived traumatic experience, loss of home and community, and the stress of adapting to a new environment (12–14). These compounding factors ultimately contribute to poorer health outcomes among refugees compared to majority populations in host countries.

### Why social prescribing may be suitable for refugee populations

Such inequities could be mitigated through systemic approaches that focus on social health and creating access to community. One of these systemic approaches is social prescribing which could be appropriate for refugee populations, but has not yet been systematically assessed. In itself, social prescribing represents a promising approach given its inherent flexibility, both in the diversity of interventions, as well as bridging multiple dimensions of wellbeing (e.g., social care, accommodation issues, financial challenges). This may align well with the multi-faceted barriers refugees experience to ultimately promote better health and wellbeing outcomes.

Social prescribing interventions may address refugee-specific barriers through several interconnected mechanisms that support improved outcomes. Social connection mechanisms could activate through referral to activities, e.g., gardening projects, mother-and-baby groups, which create opportunities for relationship building, potentially reducing isolation. Navigation and access mechanisms may activate when link workers support newly arrived families access unfamiliar health and social systems, promoting access and engagement, e.g., explaining how to register with primary health care services. Empowerment mechanisms could activate when participation in skills-building activities, e.g., language groups, builds confidence and agency for individuals whose capacities for self-determination have otherwise been severely constrained. Understanding which mechanisms activate under which contextual conditions for which individuals or groups requires systematic synthesis. Related relevant questions could consider for instance whether cultural bridging is more critical during early settlement stages, or whether navigation support needs differ between newly arrived or more established individuals.

### Gaps in current research and the need to consider relevant wider evidence

Synthesizing the evidence to support more appropriate service delivery for refugee populations remains a critical research gap, deserving of a systemic response (15). The evidence base for social prescribing is in general fragmented and exceptionally context-dependent, which poses known challenges to generating evidence-based recommendations (5, 16). However, research demonstrates that tailoring social prescribing for specific populations improves effectiveness, as shown in interventions for people with autism or older adults populations (17–19). A recent scoping review assessing social prescribing for migrant populations in the UK illustrates both the need for and challenges to synthesizing comparable evidence (20). The authors found that findings were affected by a lack of needed evidence and concluded with a call for evidence to better understand migrant population needs (20). The authors used an inclusivist approach to define ‘migrants’, which means that all migrant populations including refugee populations were considered. However, results for specifically refugee populations were not differentiated in final discussion and findings. The review emphasized that more evidence was specifically needed to understand the additional roles link workers took on to support migrant populations, in comparison to majority populations (20). Based on this recent research, we expect that evidence on social prescribing for refugee populations will be scarce, fragmented, often informal and context-dependent and that interventions may function differently to those for majority populations.

Given the expected sparsity of direct evidence, relevant wider evidence may be available from social capital-based interventions as well as formal social prescribing interventions. These are two distinct but related interventions types, whereby social prescribing interventions are defined by a specific operational model: referral (often from health services) to social activities or support (21). These interventions often involve a formal (self-)referral mechanism, leading to connection to community-based non-clinical resources, and typically include a link worker or navigator role who leads an initial individual needs assessment and facilitates the referral process. By contrast, comparable social capital-based interventions will encompass broader intervention forms typically involving referral to social activities or support (22). These interventions share functional characteristics with social prescribing, such as creating new social contacts and providing supported signposting, but may lack a formal referral pathway, an individual needs assessment and individually tailored referral suggestions. The key distinguishing criteria are referral source and formalization, process, and terminology. Many community-based programs for refugees may operate through comparable mechanisms without being labeled as social prescribing. Openness to this wider evidence base could generate insights into effective approaches to social prescribing practice in the absence of other evidence.

### Participants, concept, and context framework

Identifying how and when such social interventions are effective, and for whom, is critical to better future social prescribing delivery. Broadly spoken, health promotion program adjustment to and efficacy for refugee communities is inadequately understood (13, 23, 24). This means that it cannot be adequately implemented in future provision, or in changes to existing systems, processes, and projects. This of course needs to be differentiated by population subgroups, rather than generalized: for instance, what is observed for women from one cultural context must not be assumed to be the same for women from a similar context in another setting.

Based on this need, the review will employ the Participants, Concept, and Context (PCC) framework to structure or compare evidence and ensure clarity in addressing the research questions (25). In this approach, participants refer to those populations engaged in the intervention, concept to the nature of the intervention delivered, and context to the (country) setting in which this is delivered (as described in Table 1). This framework is particularly relevant as it enables a systematic focus on the key dimensions of intervention applicability and effectiveness in this complex field.

**Table 1:** Participants, concept, and context framework.

Participants: Populations engaged in the intervention	Concept: Nature of the intervention delivered	Context: Country and operational settings
e.g., refugee populations (including asylum seekers and forcibly displaced persons)	e.g., social prescribing interventions	e.g., any country setting, delivery settings with specific exclusions

This review will therefore systematically evaluate the evidence on the effectiveness of social prescribing interventions for refugee populations, focusing on what approaches work, for whom, and under which circumstances. Additionally, the review will assess social capital-based interventions operationalized in comparable ways to social prescribing for additional insights. By addressing these questions, the review aims to map intervention methodologies, identify key mechanisms and contextual factors, and inform tailored program delivery to better support refugee populations.

### Objectives

The primary objective is to identify evidence for what approaches to social prescribing for refugee populations are effective or “work,” for whom, and under which circumstances. This evaluation will consider both evidence resulting from defined social prescribing interventions (RQ1), as well as that resulting from social capital-based interventions which are operationalized in comparable approaches to social prescribing (RQ2).

*RQ1*: What approaches to social prescribing focused on refugee populations work, for whom, and in what circumstances?

*RQ2*: What additional insights can social capital-based interventions provide about which approaches with refugee populations may work, for whom, and in what circumstances?

Evidence from both research questions will be synthesized together through integrated analysis. Rather than maintaining separate synthesis streams, included studies will be mapped to identify intervention families (e.g., groupings of comparable studies) based on operation characteristics rather than intervention labels. This allows formal social prescribing interventions (RQ1) and comparable social capital-based interventions (RQ2) to be analyzed within the same families where they share functional approaches. Program theories (e.g., specific hypotheses) will be developed across both evidence streams, clarifying how mechanisms are assumed to operate within contexts to produce outcomes regardless of whether evidence derives from interventions labeled as ‘social prescribing’. Final synthesis outputs will clearly indicate whether program theories are supported by formal social prescribing evidence, comparable social-capital interventions, or both, maintaining transparency about evidence while maximizing learning from the available evidence.

## Methods

### Realist review methodology

This review uses realist review methodology to explore the mechanisms underlying social prescribing and social capital-based interventions and the context in which they are implemented (25, 26). This approach has been used in previous social prescribing reviews and is designed to address complexity in health services where interventions act within complex social systems (6, 27, 28). Broadly, it also reproduces some of the functions known from scoping reviews, such as examining a broad area of knowledge, identifying gaps, emerging trends, and examining conduct of research (29). A key element of a realist approach involves assessing different layers of social reality, whereby the same intervention may succeed, fail or lie anywhere in-between depending on its contexts and implementation (6, 25). Realist review enables mixed-methods evidence assessment, synthesizing heterogeneous evidence assessment pragmatically by pursuing questions of relevance and rigor rather than focusing solely on evidence quality hierarchies (6, 25). This allows meaningful synthesis across diverse populations’ experiences, contextual factors, and evidence forms.

Rather than measuring whether interventions work based on defined metrics, realist review examines why they worked, how they might be operationalized in different contexts, and for whom they are effective (25, 26). The main focus of this review’s enquiry is understanding Context-Mechanism-Outcome interactions (CMO): how intervention components activate mechanisms (participant reasoning and responses) within particular contexts to produce outcomes (25). For example, when evaluating if social prescribing reduced loneliness for unaccompanied refugee minors, realist review examines whether mechanisms changed for participants (e.g., have capacities for social agency changed?) within their lived contexts (e.g., are there a wide range of accessible social activities?). Synthesis involves exploratory assessment to identify program theories, which are hypotheses about how interventions work, then systematically examines mechanisms and their interactions with contexts (6, 26). In this review, theory-building supports identification of intervention families before assessing CMO relationships and developing program theories to provide actionable insights (30).

### Rapid realist reviews

Realist reviews require sustained and considerable resource investment over a long span of time, which may outstrip resources available and require commitment to a lengthy process. While preserving the key components of realist methodology is important, ‘Rapid Realist Review’ methodologies have developed in response to the challenge of producing high quality knowledge syntheses at speed for policy questions (30). Rapid review forms have been critiqued for the compromises seen with these methodologies and for the validity of final findings, but remain a pragmatic approach that balances real world demands with ideal data synthesis (30, 31).

The decision to conduct a rapid realist review here is guided by three criteria: timeliness, policy relevance, and feasibility. First, the most recent UK review of social prescribing for migrants was commissioned through a national call for evidence and highlighted the lack of refugee-specific data (20, 32). Second, the topic is highly policy-relevant, as demonstrated by the recent funding of the Horizon Europe project Social Prescribing for Health and Wellbeing (2025–2029), which will investigate social prescribing for refugees, migrants, and other groups (33). Finally, rapid realist reviews prioritize the policy and practice relevance of final findings, concentrating on creating and sharing immediate and actionable insights (30). By systematically synthesizing existing international evidence, our review will provide a timely and policy-relevant foundation to directly support and inform these ongoing initiatives.

Stakeholder engagement is an important element in rapid realist reviews to inform and ensure relevance of final findings (25, 30). In this review, question-finding was informed by initial stakeholder conversations that evolved into a series of expert interviews (34). Ongoing stakeholder involvement will take the form of targeted engagement with an external advisory board composed of practitioners and researchers working with refugee populations, who will be consulted at key stages such as sense-checking emerging findings and final program theories. This ensures that while our design is adapted to doctoral resourcing, it retains the critical stakeholder perspective that is central to realist and rapid realist methodology.

Our approach will follow the Realist And MEta-narrative Evidence Syntheses: Evolving Standards (RAMESES) publication standards (30, 35), balancing timeliness with methodological rigor. Rapid realist reviews have precedent in social prescribing research (19, 36), providing an established framework for producing actionable insights within shorter timeframes. Using a rapid realist review approach therefore provides a framework whereby this research can reasonably be completed and shared for a wider audience at speed, in order to extend previous research and support ongoing international initiatives (20, 32, 33).

A detailed comparison of methodological adaptations from traditional realist review to the rapid approach used in this study is provided in Supplementary File 1: Rapid review process summary table. Each adaptation is justified based on timeliness, policy-relevance, and feasibility while preserving the core realist principles of theory-driven synthesis and CMO analysis. The methodology proposed here necessarily involves pragmatic trade-offs compared to traditional exhaustive approaches, with inherent risks of selective evidence capture and interpretation bias. To mitigate these limitations, this review will implement multiple safeguards:

1. Calibration procedures: Dual screening of 15% of records with structured discussion and resolving ambiguous cases before single-reviewer screening to establish consistent application;
2. Exhaustive citation chasing: Systematic citation chasing applied to all included studies and reviews to reduce risk of missing key evidence;
3. Triangulated search approaches: Combination of conventional database searching and tailored grey literature methods to capture diverse evidence and reduce dependence on any single search method;
4. Transparent stopping rules: Documented decision criteria for grey literature searching provide audit trail and justification for search scope limitations;
5. Cross-verification of extraction: Independent cross-checking of data extraction by second reviewer reduces individual interpretation bias in coding;
6. Stakeholder involvement: Expert advisory board review of program theories grounds findings in practice experiences and helps reduce risk of potential misinterpretations or overreach;
7. Comprehensive documentation: Detailed supplementary materials promote transparency in process and peer assessment or reproduction of synthesis.

While rapid review methodology inherently applies constraints compared to multi-year traditionalist realist reviews, these mitigation strategies are planned to balance feasibility with methodological rigor to support trustworthy synthesis that can inform policy and practice (30).

### Review design

We will follow the basic principles of realist review, namely theory-driven synthesis through the analysis of CMO configurations (25), but explicitly adapt these within a rapid realist review design (30). In practice, this means applying a purposive and resource-sensitive search strategy, involving stakeholders at key stages rather than throughout the full iterative process, and focusing on generating timely, policy-relevant insights (6, 27). Our reporting will follow the RAMESES standards for realist syntheses (35), particularly regarding the development, testing, and reporting of program theories. Our approach will embody the retroductive reasoning central to realist methodology, where we move iteratively between data and theory to build explanatory accounts grounded in the evidence while drawing on theoretical insights to make sense of patterns.

There are multiple methodological phases planned for the review, beginning with (RQ1) the identification of those interventions self-identified as social prescribing, focused on refugee populations in terms of their model alignment, forms, and components. An additional enquiry (RQ2) will assess evidence from social capital-based interventions for additional insights into what may work for these populations focused on their model alignment, forms and components.

Following this identification and assessment of included studies, in the following phase, families of interventions and program theories will be identified to evaluate which factors indicate whether an intervention may be successful in engagement, adherence and outcomes (i.e., may ‘work’ or ‘not work’). These program theories are expected to take the form of ‘if-then’ logic statements for intervention families, describing how social prescribing models for refugee populations are expected to work, and underpinned by mechanisms bridging the context and outcome. This process will consider the role of specific population (‘for who’) and contexts (‘under which circumstances’) in its mapping of evidence.

### Eligibility criteria

Examples of studies that would be meet inclusion criteria include all quantitative or qualitative field studies assessing an active intervention, program, project or reports of the same. Evidence without this active intervention element will only be retained for possible snowballing if considered highly relevant. In this case a review of social capital-based interventions for refugees, and of social prescribing for migrants, would be included (20, 22), whereby a review of area level social fragmentation and schizophrenia would be excluded (37). There will be cases of background articles which are highly relevant to the subject, which will still be excluded based on this requirement – for instance, considering the role of housing in social and community capital for farming and immigrant families (38). This review will consider (digital) evidence available in English, which although considered the common language for scientific work will still likely lead to potential evidence published in other languages being excluded (e.g., articles published in German or Spanish considering migrant social capital and information availability) (39, 40).

Using the Participants - Concept – Context framework for this review gives greater clarity to the research questions and objective framing for the review (29), specifically what are the inclusion and exclusion criteria. These are summarized in Table 2.

**Table 2:** Initial inclusion and exclusion criteria.

PCC framework and study type	Inclusion criteria	Exclusion criteria
Participants	Refugee populations, asylum seekers, forcibly displaced persons (any age, gender, stage of settlement) Internally displaced persons (considered as a separate category)	Studies where <50% are refugees Studies focusing only on return/re-acculturation in country of origin Service providers without direct refugee participation
Concept	Social prescribing interventions: referral from health services to activities Social capital-based interventions operationalized in comparable ways (e.g., peer support, supported signposting)	Interventions confined to closed communities without intervention components (see clarification) Interventions are not operationalized comparably Studies lacking sufficient methodological detail to understand mechanisms Further exclusion cases (see clarification)
Context	Any country context Formal search: Published between 2014–2024 Publications in English	Non-English publications
Study type	Quantitative, qualitative, or mixed-methods empirical studies Relevant grey literature (project reports, evaluations)	Purely theoretical/conceptual papers Background articles without active intervention elements

#### Participants

In this framing, ‘participants’ refers to refugee populations whereby there may be great variation in constellations, ages, and point of intervention (e.g., how long the group have already been in the host country). Refugee populations include those identifying as refugees, asylum seekers, and forcibly displaced persons, without a defined time period of settlement in the host country. Internally displaced persons, i.e., populations who have been forced to move within their country of residence will be considered as a separate category of evidence, depending on whether this evidence is found. Institutions and service providers are considered separately and are therefore not critical to inclusion criteria. Specifically excluded from consideration are studies where the minority of participants (i.e., <50%) are defined as refugees or asylum seekers and studies focusing on re-acculturation for refugee populations, after a return to their country of origin or original place of residence.

A common issue is likely to be population definition, as many already identified studies in the trial screening process often refer to both migrants and refugees. In these cases, this review will define refugees within migrants groups using a residualist approach – a migrant is a person who moved countries for reasons excluding war, natural disaster and persecution (41). A person who migrated based on these reasons was forced to migrate, and is defined here as a refugee. For studies with mixed populations, a specific protocol will be implemented. Studies reporting ≥50% refugee participants will proceed to full-text screening. During full-text review, disaggregated results for refugee subgroups will be prioritized for extraction where available and studies without disaggregated results will not be included. Where refugee-specific applicability is unclear from methods or discussion, studies are discussed between reviewers and assessed based on likely transferability of identified mechanisms to refugee contexts. All inclusion decisions for mixed-sample studies will be documented with rationale in screening records to ensure transparency and consistency.

It is expected based on dynamics of global knowledge production that the majority of evidence will be identified from formal publications or grey literature from the minority world (global north). The evidence base may not reflect lessons from the environments, realities, or knowledge capital for the majority of persons affected by forced migration. These are overwhelmingly internally displaced persons, or refugees hosted by neighboring countries in the majority world (global south) (42, 43). This is then a partial capture of all contexts where social prescribing and social capital building takes place for refugee populations, which is a relevant limitation for this review and for social prescribing systems more generally.

#### Concept

The intervention strategies for social prescribing or social capital-based approaches will be further defined via referral sources and intervention content as responds to RQ1 and RQ2. These will be categorized as either: (RQ1) a referral from health services to social activities/support, here defined as social prescribing evidence, based on operational mechanisms defined the Delphi study by Muhl et al. (21); or (RQ2) a (self-)referral from any other source to social activities or support in the context of a social capital-based intervention, where the social activities or support could be operationalized as social prescribing (e.g., new social contacts, supported signposting to group or communal activities, or designated new functions in existing social relationships).

There are of course challenges to pursuing this conceptual richness with such an open approach to considering broad forms of social interventions. The most challenging criterium in this regard concerns assessing mechanisms, responding to the question of whether this intervention form could be replicated for social capital-based evidence. Some studies will not meet this criterium because the social capital building takes place inside closed communities without intervention components, organized and operated on an individual level. A good example of this borderline case is “Social Capital and Rural Health for Refugee Communities in Australia”, a qualitative study as part of a larger project, focusing on the relevance of social ties for refugee health (44). Although undoubtedly highly relevant and related to the topics of this review, the process by which individual refugees were socially welcomed by members of their own communities (or via institutions, such as through church communities) firstly does not meet the criteria above for an intervention element or component. It does not mimic a referral from any source to an institution, which also means there is no detail to understand how this could be replicated and expanded in a systems approach. Secondly and relatedly, the detail of how these integration processes functioned is lacking, which means the study does not relay the conceptual richness which would be critical to this review. It’s not possible to derive a methodology from the data available to understand how this could operationalized in a systematic way, how it could be replicated and expanded. Another unclear case which would however be included is “Building Social Capital Through a Peer-Led Community Health Workshop: A Pilot with the Bhutanese Refugee Community” (45). This paper details the development of a Community Health Workshop with community leaders, which although being a one-off event (not typical for social prescribing referrals), ran for nearly a week and covered multiple topics including mental health and wellbeing in the population language Nepali. It functioned as part of a larger community-based participatory research project focusing on wellness partnership building, culturally competent training of refugee community leaders and service providers, and a final peer-to-peer community health intervention. The paper is conceptually rich in its methodological description of what was delivered and how the workshop was prepared, with an accompanying paper available to allow the replication of training elements, and the overall project structures and processes (46, 47). Although not in itself meeting a strict definition for social prescribing, the workshop and related project’s outputs meet the criterium for understanding how its mechanisms worked, and would therefore be included as a social capital-based example study to enrich final recommendations.

In summary, as this review assesses whether evidence from social capital-based interventions could provide additional insights for social prescribing approaches for refugees, the final included study selection will be broader and more diverse than that typically seen for social prescribing. This challenge requires additional care and attention in the screening and selection process, to consider what should be included, what should be excluded, and how that boundary will be defined. For instance, due to the nature of evidence identified by the broad search, specific case-based exclusion criteria for the concept framework were required once all material had been sighted, as seen in Table 2. This process will involve intense discussion and reflection on the part of key reviewers involved in both screenings, and will likely by adjusted in response to the final evidence seen.

#### Context

It is expected that a broad range of contexts will be described in minority countries (global north). Given the need to exploratively assess all available approaches which may have insights for social prescribing practices, all contexts for delivery will be considered where evidence meets inclusion criteria for participants and concept above. In the formal search, research publications within the last 10 years (2014–2024) and published in English will be considered.

### Clarification of specific exclusion cases

To support consistent application of exclusion criteria, the following categories are explained in more detail and specific exclusion cases are described:

Closed communities without intervention components: Research occurring exclusively within pre-existing, bonded social groups (e.g., church congregations, ethnic community centers, informal friendship networks) without formal intervention structures are to be excluded. Criteria to assess includes documented intervention structure or protocol that could be adopted by other settings, sufficient methodological detail to understand core components and mechanisms, evidence of delivery beyond purely organic unstructured social connections.

Residential interventions: Interventions where participants live in institutional or group residential settings (e.g., residential treatment programs) are excluded because their operationalization differs fundamentally from community-based social prescribing models. In this case, community-based social prescribing models assumes participants maintain independent living arrangements and access activities in community settings, whereas residential interventions operate within bounded institutional contexts with different mechanisms of social connection and support.

CBPAR/PAR focused interventions: Community-Based Participatory Action Research studies focusing solely on intervention development or co-design processes without reporting on final intervention methodology and delivery, participant outcomes, and mechanisms of effect are excluded as they lack the operational detail needed for realist synthesis.

Digital interventions without social component: Digital or self-help interventions with no interpersonal or community-based social element (e.g., purely app-based mental health tools, online psychoeducation without peer interaction) are excluded as these are not operationalized with a social component and hence are not comparable to social prescribing.

Refugee-as-provider studies: Studies examining refugees as mentors, befrienders, or peer supporters without reporting on the experiences or outcomes of refugee mentees or befriendees are excluded because the mechanisms and outcomes for refugee participants as service users (rather than providers) are central to this review’s focus.

### Definition of terminology

Linked to the above, the terminology for ‘social prescribing’ is primarily driven by practice in the UK, with only recent take-up in other countries (1, 48). This poses challenges in the search for international evidence, where a variety of terms must be considered to identify all evidence that may describe delivery models that function in ways similar to social prescribing, despite not being named as such. We are aware that social prescribing has a diverse and diffuse practice as well as terminology, and will maintain a particular focus on things that self-identified as social prescribing and meeting that definition within this review. At the same time, we will conduct broader searches for social capital-based interventions to capture additional learning we think might be relevant in building our realist understanding. On this basis, two sets of terms are planned to respond to the research objectives in the search, whereby the second will focus on interventions broadly aligned to social-capital building, but which are operationalized similarly to social prescribing. At the same time, both sets of terms are also likely to identify ‘informal’ evidence not submitted in the form of academic publications. This reflects the practical reality that creating social connections to others for support is, after all, a fundamentally human drive. This evidence will also be considered to the fullest extent possible for resulting program theories, although it may not be detailed enough for fuller program mechanism consideration.

### Search strategies

The search strategy will be developed by the project lead and reviewed in discussion with the wider team through the screening and coding processes. The search will first address RQ1, evidence relating to social prescribing, then RQ2, evidence derived from social capital-based interventions with operationalization comparable to social prescribing. Initially a pilot coding test will be run to assess proposed methods against the results found, with adjustments made iteratively to search strategies and coding as needed. This iterative refinement process includes:

1. Testing term precision: All terms will be tested for excessive noise (e.g., “social isolation” generating 179 PubMed results with many studies not directly relevant to intervention research) were refined by testing different field restrictions (MeSH terms, Title/Abstract versus All Fields) to improve precision while maintaining sensitivity to capture relevant intervention studies;
2. Optimizing database-specific syntax: Search strings were adapted to each database’s structure (e.g., Embase ‘/exp./’ for exploded terms, Web of Science ‘ALL=’ versus ‘TS=’ or ‘AB=’ field specifications, SCOPUS ‘TITLE-ABS-KEY’ for focused retrieval) based on initial result assessments;
3. Expanding social concept terms: Social capital terms were incrementally expanded (e.g., adding ‘mentoring’, ‘peer support’, ‘bridging’) when searches in individual databases identified relevant studies;
4. Excluding negatively connotated concepts: After testing all individual terms and completed strings, negatively connotated concepts that may still have been linked to intervention studies (e.g., ‘social discrimination’, ‘racism’, ‘social isolation’, ‘ostracism’, ‘social marginalization’, ‘social stigma’) will be removed if these produce excessive noise.

The inclusion of broad social-capital terms such as ‘interpersonal relations’, ‘social integration’ and ‘community participation’ reflects the exploratory nature of this review and the absence of established terminology for social capital-based interventions. While these terms occasionally generated higher volumes when combined with refugee terms (e.g., ‘social integration’), piloting demonstrated they captured conceptually relevant interventions not identified through narrower terms alone. The planned three-block Boolean structure (refugee terms AND social terms AND intervention context terms) will provide the necessary specificity, with the ‘intervention context’ block filtering results to intervention research rather than purely theoretical or epidemiological research. Term breadth is deemed necessary given the fragmented evidence base, diverse labeling of comparable interventions across contexts, consistent with a broad scoping approach.

The two sets of terms to be used for these phases will focus on the function of referral to community or social settings to support health and wellbeing outcomes for literature from 2014–2024, and are combined in the social concept terms block below. Identified studies will be managed and screened using the review program Rayan for reviewing purposes. Electronic databases to be searched in both cases include:

- PubMed;
- Web of Science;
- Embase;
- CINAHL;
- SCOPUS;
- PsycInfo.

To illustrate in the case of PubMed, the following search string was used consisting of three blocks (refugee population + social concept + intervention context) with a limit to articles published between 01.01.2014–28.10.2024 (date of search):

*Refugee terms block*: ((refugee*) OR (asylum seeker*) OR (displaced person*) OR (“forced migrant”) AND.
*Social concept terms block*: (“social prescribing”[All Fields] OR “social capital”[All Fields] OR “interpersonal relations”[All Fields] OR “social integration”[All Fields] OR “social cohesion”[All Fields] OR “social interaction”[All Fields] OR “social skills”[All Fields] OR “social relationships”[All Fields] OR “social inclusion”[All Fields] OR “social adjustment”[All Fields] OR “community participation”[All Fields] OR “community involvement”[All Fields] OR “community resources”[All Fields] OR “peer support”[All Fields]) AND.
*Intervention context*: (“review”[All Fields] OR “intervention”[All Fields] OR “project”[All Fields] OR “program*”[All Fields] OR “trial”[All Fields] OR “study”[All Fields]).

Full search strings for all six databases, including database-specific syntax and field specifications, are provided in Supplementary File 2.

A great deal of literature for the topic will also be found in grey sources as part of a supplementary search strategy. From a previous similar review, comparing studies identified via a standard ‘conventional approach’ to those found via extensive supplementary searching demonstrated that evidence found through the latter made the more substantive and unique contribution to final findings (49). Authors from the review emphasized the need to consider sequential order of study identification methods, giving them a different prioritization in terms of effort and time depending on the relative anticipated value (49). Additional searching methods to complement the database searches will therefore be implemented and developed iteratively through this review process, based on suggestions for ‘A Tailored Approach’ from Cooper et al. (50). This will include snowball procedures for citations from included studies (‘citation chasing’), hand searching the social prescribing sub-group on the Future NHS collaboration platform (‘hand searching’), and contacting institutions delivering social prescribing for project reports, research centers, practitioners, or other relevant stakeholders (‘contacting study authors’). Web hits will also be considered, both using Google and Google Scholar results (‘web searching’ and extending the conventional ‘bibliographic database searching’).

Stopping rules will be applied consistent with rapid review principles prioritizing feasibility in balance with reaching conceptual saturation from the potential evidence base:

1. Google and Google Scholar searchers: Searching will be stopped after scanning the first 100, 250, or 500 hits where further scanning yields diminishing returns in terms of relevant new material;
2. Organizational outreach: Direct outreach to organizations, networks, and practitioners will be conducted using a systematic search of UK charity registers and snowballing from recommendations from responders;
3. Citation chasing: Citation chasing will be applied systematically to all included studies at full-text screening and to all relevant reviews;
4. Google Alerts: Google Alerts will be set up for ongoing monitoring of new publications, with a defined endpoint corresponding to the commencement of final analysis.

These stopping criteria reflect the pragmatic approach of rapid review methodology, balancing breadth of evidence coverage with the need for timely completion to inform policy and practice.

**Figure 1:**
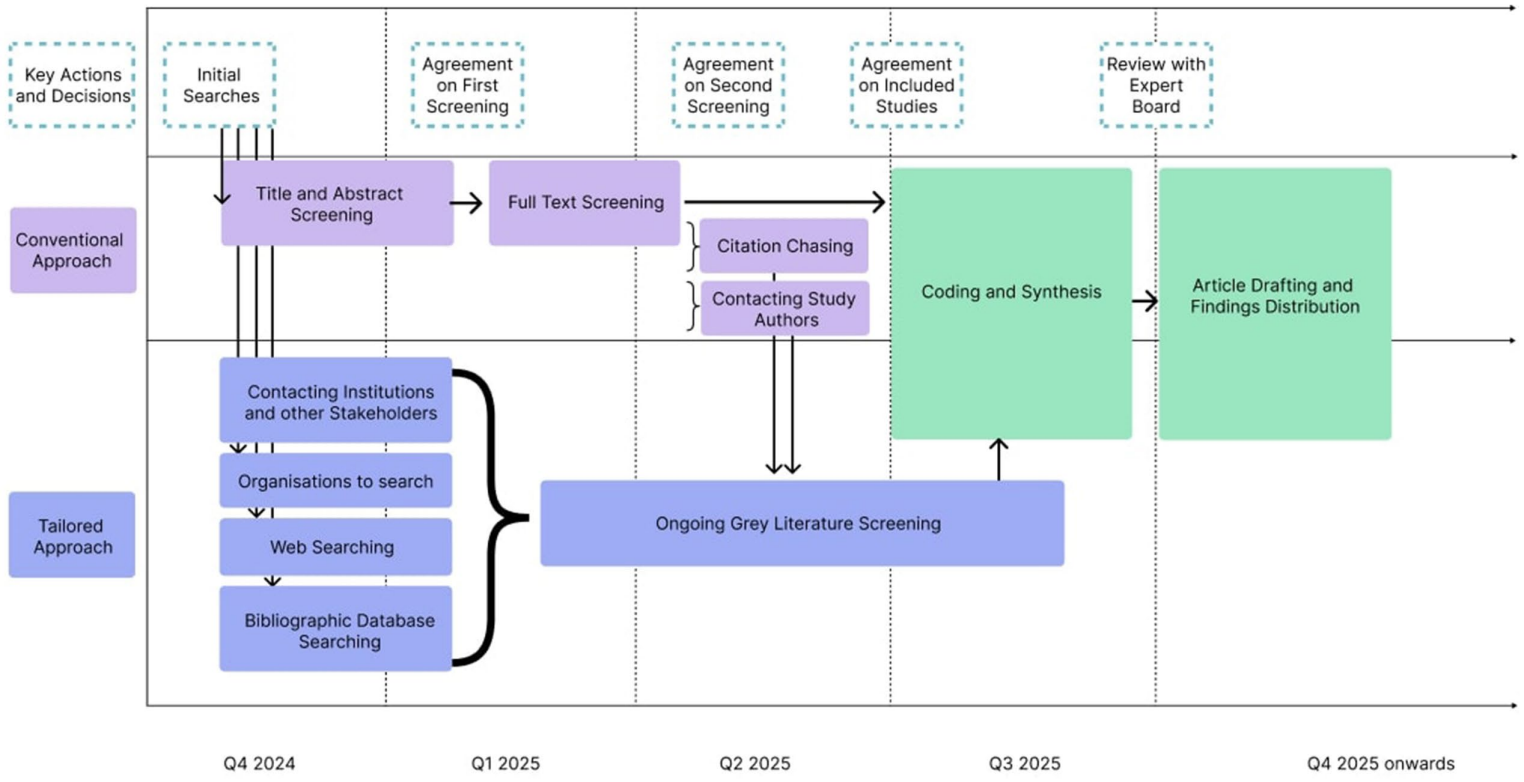
Overview of the timeline and search methods.

Non-academic stakeholder materials (including service evaluation reports, organizational briefings, community program reports) will be handled systematically through targeted requests to refugee-serving organizations based on a systematic search as described above, as well as targeted approaches to relevant institutions. Consistent screening with application of the same inclusion criteria as for peer-reviewed literature, equivalent appraisal using the same frameworks; and transparent documentation of source, document types and rationale for inclusion or exclusion also supports systematic handling. This approach recognizes that practice-based evidence from community organizations is central to understanding real-world implementation, consistent with the emphasis in rapid realist review methodology on capturing diverse evidence forms (30, 50) (see Figure 1).

### Data management

The included files from the search will be cleaned for any duplicates and managed using Rayyan using a bespoke project, to which all three reviewers have access and editing rights. Search summaries will be recorded and managed in Rayyan. Screening processes will be documented and presented using a PRISMA 2020 flow diagram to ensure transparency of study selection (51). Reporting will follow the RAMESES publication standards for realist syntheses (35), which are more appropriate for realist review methodologies. Rayyan will be used for all data management until final included studies are agreed, at which time data lists for all stages of screening will be exported for reference and results coding for included studies will be completed using Excel.

### Selection of studies and assessment

A sample will be created from the results before screening, whereby two members of the publication team will review 15% of the total titles and abstracts following precedent from a similar review. Following this sample screening, both researchers will discuss inclusion criteria based on the screening process and resolve any conflicts with another neutral party, the third reviewer (52). This calibration exercise serves to establish shared understanding of inclusion/exclusion criteria, develop case-based decision rules, and resolve sources of disagreement to ensure consistent application. Specific decision rules for subjective criteria (such as assessing whether this is an intervention) are documented in screening notes, with exemplar cases retained for reference during the remainder of screening. Following this exchange and agreement on case-based exclusion criteria, the remaining articles will be screened by one reviewer in the second phase of title and abstract screening. In cases where no title and abstract are available (e.g., project reports, briefings), screening will be conducted using summary pages, executive summaries, briefings, introduction screenings, or specific searches using key terms to review relevance from longer documents. This standardized approach to grey literature screening helps mitigate selection bias by ensuring consistent application of inclusion criteria across diverse document types. Where the documents seem to fit inclusion criteria under either RQ1 or RQ2, these will be included for a longer full text screening.

### Appraisal of relevance, richness, and rigor

Following completion of full text screening, all potentially includable evidence will be appraised for its relevance, richness, and rigor, in keeping with realist review approaches and existing practical guidance (26, 53, 54). This appraisal process serves to prioritize studies that will most meaningfully contribute to program theory development while maintaining transparency about evidence quality. In this appraisal process, relevance is defined by whether it informed development or testing of program theories, richness is defined by how theoretically informed and detailed the methods are, in order to understand mechanisms, and rigor is defined by the trustworthiness of methods and plausibility of findings. A hybrid appraisal approach was used to ensure both qualitative and quantitative evidence were considered within the same framework.

Relevance will be assessed by determining whether and how each study informs the development or testing of program theories related to social prescribing or comparable interventions for refugee populations. Studies will be classified as having high, medium or low relevance. High relevance refers to studies which directly address refugee populations in social prescribing or highly comparable social-capital interventions with clear mechanism descriptions. Medium relevance refers to studies which address refugee populations in related interventions, with some transferable insights. Low relevance refers to studies with tangential relevance or limited transferability. Only studies assessed as having high or medium relevance will be prioritized for final inclusion.

Richness (in terms of conceptual depth) will be assessed based on how theoretically informed and methodologically detailed the study is for understanding mechanisms. The “rich, thick, thin” classification will be applied in terms of conceptually rich studies, conceptually thick studies, or conceptually thin studies. Conceptually rich studies include detailed descriptions of intervention mechanisms, participant experiences, contextual factors, and theoretical framework. Conceptually thick studies will have moderate detail on mechanisms and context but with less theoretical depth. Conceptually thin studies will have minimal description of mechanisms or context, typically being outcome(s) focused with limited process information. Conceptually rich and thick studies will be prioritized for inclusion, as they provide the detailed mechanism descriptions that is key for realist synthesis. Agreement for classification of studies will be applied collaboratively through discussion between two reviewers after assessments are primarily carried out by one reviewer.

Rigor (in terms of methodological quality and trustworthiness) will be assessed using appropriate standardized tools, dependent on the study design and following the example of previous realist review work (54). Randomized controlled trials were assessed using the CASP RCT checklist, qualitative studies using the CASP qualitative checklist, quantitative non-randomized studies using the NIH quality assessment tool, and mixed-method studies using the MMAT mixed methods appraisal tool (55–58). These assessments will evaluate the trustworthiness of methods and plausibility of findings, with particular attention to how well studies describe their intervention implementation, participant contexts, and outcome measurement.

### Appraisal process and bias mitigation

The appraisal process will be conducted primarily by one reviewer, with all results being subsequently reviewed and verified by a second reviewer. This dual-review approach helps to mitigate individual bias in quality judgments. Where discrepancies arise in relevance, richness, or rigor ratings, reviewers will discuss the evidence and reach consensus, with consultation of a third neutral reviewer if needed. To further minimize selection bias, transparent bespoke criteria in line with the descriptions above will be applied consistently across studies. Decisions will be documented, including classification and rationale. Both academic and grey literature will be appraised using the same framework, with the aim of reducing the risk of privileging peer-reviewed sources. Studies with findings that challenge emerging patterns will not be excluded solely on that basis to ensure diverse perspectives are retained.

The final set of included studies will be those judged to have sufficient relevance, richness, and rigor to contribute meaningfully to program theory development, while acknowledging that pragmatic decisions about study prioritization are needed.

### Data extraction

In order to ground any resulting recommendations and to provide greater clarity concerning intervention families, their forms, and contexts, capturing wider information about the methodologies, concepts and contexts will be essential in the final data extraction for included studies. This includes (and is not limited to): populations involved; setting information; timespan for the intervention; intervention type; institutions or service providers; financing; recruitment and engagement strategies; translation or language support; staffing incl. Training and support; outcome measures; evaluation procedures; and non-intended effects. This will be completed using an inductive approach to designing a final coding book, whereby the original data categories can be extended based on evidence from the final included studies. This would take place based on exchange between two researchers, whereby one would complete an initial coding that will be cross-checked by the second, for all final included studies. Data management and coding will be supported by structured Excel templates with documented version control to ensure systematic organization of extracted data, enable transparent tracking of coding decisions, and facilitate retrieval of evidence supporting each program theory.

As examples of extracted data: under ‘delivery modes’, we will code whether interventions use peer facilitators, link workers, group-based facilitation, or hybrid approaches. Under ‘enablers’ we will code practical supports such as interpretation services, transportation assistance, or childcare provision and child friendly spaces, as well as relational factors such as delivery in trusted community locations or community-led staff members. Under ‘outcome measures’ we will document both quantitative indicators (e.g., validated mental health scales, service metrics) and qualitative outcomes (e.g., reported sense of belonging, navigation confidence, social connectedness). Focuses for data extraction will also be informed by interviews with experts working with refugee populations in the UK in an affiliated project (34). This approach ensures both standardized categories are applied, based on wider stakeholder input, and retains flexibility to capture emerging themes specific to the material.

### Data synthesis

A key focus for this review will be mapping the conceptual methodologies of interventions identified and included after the full text screening. As becomes clear from assessment of the background and population need, very little is known regarding refugee populations’ experience of social prescribing or social capital-based interventions. This review then performs a collating function to gather diverse and heterogenous evidence, asking the question which evidence could give valuable insights to designing system approaches and policy.

### Development and validation of program theories and CMO configurations

The development of Context-Mechanism-Outcome (CMO) configurations and program theories will follow an iterative, multi-stage process designed to ensure methodological rigor while maintaining transparency in how conclusions are derived from the evidence base. This process is structured according to established realist methodology (25, 35).

#### Stage 1: identification of intervention families

Following initial data extraction, all included interventions will be systematically mapped according to their operational characteristics, theoretical underpinnings, and support infrastructures. This mapping process will consider:

- Primary delivery modes (e.g., link worker/case management, peer-led, group-based, structured program);
- Primary and secondary intervention focuses (e.g., mental health support, navigation/access, social connection, skills-building);
- Intervention features (e.g., referral-led, cultural adaption, trauma-informed practices);
- Enablers, both practical/material (e.g., interpretation, transport, child-care) and structural/relational (e.g., trusted locations, unstructured social time, onward referrals).

Based on this mapping, interventions will be grouped into ‘families’, distinct groupings of interventions that function comparable despite potentially different labels or setting adaptations. Family membership will be determined primarily by operational mechanisms, recognizing that similar approaches may be conceptualized differently across contexts. A decision tree will guide this allocation process, developed iteratively by the reviewer team and refined through discussion. This family-based approach reflects the rapid realist review emphasis on identifying intervention-outcome patterns within specific parameters.

#### Stage 2: distinguishing mechanisms from intervention components

A key analytical task involves distinguishing mechanisms (the underlying reasoning or responses of participants that lead to outcomes) from intervention components (the resources or activities provided). Following Dalkin et al.’s conceptualization (59), we will differentiate between:

- Intervention components (mechanism resources): The tangible elements and offerings of the intervention, so what is provided or made available to participants (e.g., provision at a trusted location, peer mentoring, interpretation services, skills training workshops);
- Mechanisms (mechanism reasoning): The cognitive, emotional, and behavioral responses these resources activate for participants, so how they interpret, respond to, and are changed by what is provided (e.g., increased sense of safety enabling disclosure, peer-modeling normalizing help-seeking, language support enabling understanding, culturally matched interactions triggering trust).

This distinction recognizes that intervention components alone do not produce outcomes; rather, they activate reasoning processes for participants that may generate change within particular contexts. This will be operationalized through structured focuses during data extraction on: what was provided (intervention components/resources); how participants responded (mechanisms/reasoning); what conditions were required for these responses (context); what outcomes resulted (outcomes).

While one primary reviewer will code for intervention components and mechanisms during data extraction, this process will be supported through detailed review and cross-referencing to source texts by a second reviewer with regular meetings to calibrate understanding and address ambiguities.

#### Stage 3: construction of CMO configurations

CMO configurations will be constructed by linking mechanisms (reasoning) to the contexts in which they activate and the outcomes they produce, while clearly documenting the intervention components (resources) that catalyze these mechanisms (25, 59, 60). For each intervention family, multiple CMO configurations may emerge, reflecting how different mechanisms operate in different contextual conditions. The construction process will involve:

1. Juxtaposing sources: Examining how different studies within and across intervention families describe similar or contrasting CMO patterns;
2. Reconciling sources: Identifying where apparent contradictions can be resolved by attending to differences in context or population characteristics;
3. Adjudicating sources: Giving greater weight to studies with richer descriptions of mechanisms or more robust outcome measurement when evidence conflicts;
4. Consolidating sources: Combining evidence from multiple sources that reinforces particular CMO patterns;
5. Situating sources: Considering the transferability of identified mechanisms across different refugee populations, intervention settings, and host country contexts.

CMO configuration evidence will be documented in supplementary materials, where possible detailing context features present, the intervention components or resources provided, the mechanism(s)/reasoning activated, the outcomes observed and supporting evidence from study IDS.

#### Stage 4: development of program theories

Program theories represent higher-order explanatory statements that theorize how and why social prescribing interventions work (or do not work) for refugee populations under specific circumstances. These will be developed by synthesizing patterns across multiple CMO configurations to generate ‘if-then’ logic statements. For example: “IF social prescribers engage with women with young children [context], THEN connecting them with community organizations that provide childcare or spaces where children are welcome [resources] removes caregiving barriers [mechanism reasoning], RESULTING IN greater likelihood of mothers’ attendance and engagement [outcome].”

Program theory construction will follow RAMESES standards for realist synthesis (35), which emphasize iterative theory-building through cycles of data examination, theory formulation, and refinement. While we are not conducting formal hypothesis testing in the positivist sense, our approach embodies retroductive reasoning, moving iteratively between empirical data and theoretical explanations to build and refine explanatory accounts that are grounded in the evidence base.

Program theories will be categorized according to their scope and focus:

- Cross-cutting theories: Applicable across multiple intervention families;
- Family-specific theories: Specific to particular intervention family approaches;
- Context-specific theories: Highlighting how population subgroups or settings modify mechanisms;
- Additional theories: Focused on key process elements or resources, including access, engagement, onward referral, failure conditions.

The development process will be iterative, with theories initially drafted by the primary reviewers based on preliminary synthesis, then refined as additional evidence is incorporated and contradictory findings are resolved through team discussion and later in exchange with the expert board.

#### Stage 5: assessment of evidence strength

Intervention families will be assessed for evidence strength to signal the overall confidence in conclusions drawn about each family’s effectiveness and mechanisms. For each intervention family, we will document the number and type of studies (how many study clusters contribute evidence, including study designs), the consistency of patterns (whether mechanisms and outcomes are consistently observed across studies within the family), and richness of mechanism description (for instance if a family is characterized by conceptually rich studies or relies more heavily on thin studies). Families with evidence derived primarily from grey literature or conceptually thin studies will be explicitly flagged, with appropriate caveats about the confidence in conclusions. Families will be explicitly discussed and validated with the Expert Advisory Board based on their practical experience.

The reconciliation and refinement of program theories will be guided by explicit criteria to ensure transparency and methodological rigor. Each program theory will be appraised for evidence strength based on two dimensions, following RAMESES guidance on assessing the rigor and relevance of evidence supporting realist program theories (35):

1. Pattern evidence: The consistency and breadth of patterns observed across studies (e.g., strong indicates clear, consistent patterns across multiple study clusters whereas weak indicates minimal or inconsistent patterns);
2. Mechanism evidence: The clarity and empirical support for underlying mechanisms (e.g., strong indicates well-described, empirically demonstrated mechanisms with clear participant accounts or robust measurement whereas weak indicates primarily theoretical or logical inferences without empirical corroboration);
3. Merging criteria: Program theories will be merged when they describe functionally equivalent mechanisms operating in similar contexts, even if labeled differently across studies (e.g., ‘peer mentoring’ and ‘befriending’ models activating similar social connection mechanisms);
4. Elimination criteria: Program theories with weak evidence strength (showing minimal pattern consistency and primarily theoretical mechanism evidence) will be flagged as identified research needs requiring validation subject to discussion with the expert advisory board;
5. Prioritization approach: Final program theories will be prioritized based on both evidence strength and relevance to social prescribing delivery in UK contexts. Theories demonstrating strong evidence and high policy relevance will be foregrounded in synthesis outputs, while theories with moderate evidence or narrower applicability will be clearly contextualized. This prioritization will be informed by the expert advisory board.

Overall evidence strength will combine these dimensions. Strong program theories demonstrate clear patterns across multiple studies with well-described mechanisms; moderate theories show observable patterns with limited mechanisms evidence or more limited scope; weak theories show minimal evidence and represent research needs requiring validation. This assessment will be documented transparently in supplementary materials for each program theory, acknowledging where evidence is limited or derived primarily from grey literature.

### Managing conflicting evidence, uncertainty, and reviewer disagreements

Where contradictory evidence is identified, the review team will first examine whether apparent contradictions can be explained by contextual differences (e.g., different refugee populations, intervention settings, or implementation features). The team will prioritize evidence from studies with richer methodological descriptions and clearer reporting of mechanisms. Where relevant, alternative CMO configurations will be presented where evidence genuinely conflicts, rather than forcing artificial consensus. All program theories with weak or contradictory evidence will be flagged as research priorities requiring further investigation and these will be discussed and validated with the expert board. All uncertainties will be explicitly signaled in the final review, including limitations regarding evidence quality, scope, and transferability. Particular care will be taken to avoid overstating causal inferences where empirical support is limited, using appropriately tentative language (e.g., ‘may suggest’) and explicitly acknowledging when program theories are based on limited or primarily theoretical evidence.

Throughout the synthesis process, disagreements among reviewers will be resolved through structured procedures:

1. Initial discussion: The two primary reviewers will first discuss disagreements to clarify whether they stem from different interpretations or missing information;
2. Re-examination of source material: Return to original studies to examine evidence in greater detail;
3. Consultation with a neutral third reviewer: Where disagreement persists, a neutral third team member will independently review the evidence and facilitate resolution.

These procedures ensure consistency in interpretation while acknowledging the inherently interpretive nature of realist synthesis.

### Analytical products and data transformation process

To ensure transparency in how conclusions are derived from evidence, the analysis will generate a series of documented products that trace the transformation from extracted data to final interpretations.

Stage 1 (Intervention Mapping) will be supported by providing the full extraction table for all included evidence and a further excel file for data synthesis. To support CMO coding, intervention components (resources) and mechanisms (reasoning) are separately documented across all products. The additional excel file for data synthesis will document delivery modes, focuses, features, and enablers for each study cluster. Stages 2 and 3 (CMO construction) will be further supported by this data synthesis file, which will include functionality to cluster similar interventions based on their characteristics. Through the use of pivot tables, various contexts, resources and outcomes will be summarized across all studies in interactive format that allows readers to reproduce and engage with source data. Stage 4 (Program theory development) will be supported by various products including files describing the intervention family decision tree, an overview of intervention families, theory-data connections for CMO configurations and initial if-then statement mapping, and evidence strength assessment tables. Stage 5 (Synthesis and validation) will be supported by products presenting expert board consultation materials, the refined program theory statements, and evidence gaps and research priorities.

### Data flow and transformation

1. Extraction: Individual study data captured in a structured bespoke template;
2. Coding: Studies coded for operational characteristics (including delivery mode and focuses), theoretical underpinnings (including intervention features) and support infrastructures (including enablers);
3. Mapping: Characteristics mapped across all studies to identify operational patterns;
4. Grouping: Studies allocated to intervention families based on comparable mechanisms;
5. Configuration: CMO patterns identified within and across families;
6. Theory-building: Patterns synthesized into explanatory program theories;
7. Appraisal: Evidence strength assessed using a two-dimensional framework;
8. Validation: Theories refined through expert stakeholder consultation;
9. Reporting: Final program theories, intervention family descriptions, and evidence gaps presented with transparency about supporting evidence.

All analytical products will be made available as Supplementary materials within the final published review, enabling readers to trace the complete analytical journey and assess the trustworthiness of interpretations. This commitment to transparent reporting should support reproducibility of our realist synthesis and provide materials for reference for future rapid review research.

### External advisory board

A final review of findings and recommendations will be made with an external advisory board based on recommendations (30). This board will be drawn together from international researchers and practitioners, specifically working with refugee populations and with experience of similar social interventions. Advisory board members will be recruited through targeted recruitment, personal networks and through contacting international refugee specific research centers and projects in this field. Board members will be selected to ensure: (1) diversity of expertise spanning academic research, frontline practice, and community representation; (2) experience working directly with refugee populations; (3) geographical diversity to represent different implementation contexts; (4) familiarity with social prescribing, social capital-based interventions, or comparable community approaches; and (5) ability to provide practice-informed perspectives that ground theoretical insights in real-world practice. To mitigate risks of over-reliance on single-context perspectives, we will ensure geographic diversity in board compositions, including representation from multiple countries and regions with differing social prescribing practice and refugee integration contexts and seek to triangulate board feedback across members with different professional roles. We will furthermore explicitly discuss areas of consensus and divergence in board perspectives during meetings and report tensions between board perspectives and the empirical patterns in the evidence.

The external advisory board will play a critical role in question formulation, priority-setting in the extraction, sense-checking of findings, and discussion of initial program theories. For instance, board members will be consulted after preliminary program theories have been developed and evidence strength assessed. Their role will include sense-checking (assessing whether program theories align with practice experience and expectations), refinement (suggesting improvements to wording for clarity and practice-relevance), validation (identifying where theories appear well-support or may overreach) and prioritization (validating which identified research needs are most critical for future investigation). Feedback from the advisory board will be systematically documented and used to iteratively refine program theories. Minutes from advisory board consultations will be maintained as part of the project archive. Key feedback points and how they influenced program theory development will be documented. Where board input substantially modifies a theory, this will be transparently reported in the final review. This process reflects the emphasis within RRR methodology on ensuring that findings are relevant and grounded in practice through stakeholder involvement, while balancing feasibility in the research context (25, 30).

## Dissemination

The final paper will be submitted to a high-impact, peer-reviewed journal, with a preference for open access for knowledge equity purposes. A short form briefing document for main findings will be produced for greater dissemination and accessibility. This briefing document with optional full-length findings will then be circulated in appropriate networks for social prescribing research and practice, as well as forced migration studies research networks where relevant. Following publication, results will also be shared at appropriate conferences in Germany and internationally. The dissemination timeline is planned as follows: journal submission within 3 months of review completion; briefing document produced following peer review of the manuscript; network circulation of review manuscript within 1 month of publication acceptance; briefing document dissemination and conference presentations within 6–12 months. This timeline reflects the policy urgency of the topic and aligns with rapid review methodology principles.

Results will guide international policy and program design through program theories that: identify relevant mechanisms, enabling adaptation to diverse contexts; identification of core components and contextual enablers that can inform commissioning frameworks; explicit research priorities that can guide evaluation design for emerging social prescribing programs internationally; and policy recommendations grounded in synthesis of international evidence. The realist approach supports transferability by explaining how and why interventions may work in distinct circumstances, rather than simply whether they work, enabling policy-makers to assess applicability to specific contexts. Briefing materials will explicitly highlight implications for policy development, commissioning decisions, and program design across different implementation contexts.

To mitigate risks of misinterpretation of preliminary program theories, all dissemination outputs will clearly distinguish between program theories with strong evidence support versus those representing identified research priorities requiring validation. Findings will explicitly be communicated in a context-dependent manner, emphasizing that effectiveness depends on mechanism activation under particular contextual conditions. Program theories will be framed as explanatory hypotheses supported by evidence patterns rather than definitive causal claims. Limitations will highlight evidence gaps, population heterogeneity from included studies, and the geographical concentration of included studies, as well as emphasizing the need for local adaptation and evaluation rather than direct intervention transfer. Briefing documents will use accessible language while maintaining precision to reduce risks of oversimplification.

## Data Availability

The original contributions presented in the study are included in the article/Supplementary material, further inquiries can be directed to the corresponding author.
