# Correction: Social prescribing for refugee populations: a protocol for a rapid realist review of international evidence

**DOI:** 10.3389/fpubh.2026.1871993

**Published:** 2026-05-15

**Authors:** Victoria Touzel, Anna-Lena Esser, Kerryn Husk, Doreen Reifegerste

**Affiliations:** 1 AG4 Prevention and Health Promotion, Bielefeld University, Bielefeld, Germany; 2 Faculty of Health, University of Plymouth, Plymouth, United Kingdom

**Keywords:** asylum seeker, realist review, refugee, social capital, social prescribing

Author Kerryn Husk was erroneously assigned to affiliation “NIHR CLAHRC South West Peninsula (PenCLAHRC), Plymouth University Peninsula Schools of Medicine and Dentistry, Plymouth, United Kingdom”. This affiliation has now been removed for author Kerryn Husk, and replaced with “Faculty of Health, University of Plymouth, United Kingdom.”

The original version of this article has been updated.

