# Supplementary material for: Social prescribing for refugee populations: a protocol for a rapid realist review of international evidence
Source: Front Public Health. 2026 Feb 16;14:1754718. doi: 10.3389/fpubh.2026.1754718 (PMC12950807; doi:10.3389/fpubh.2026.1754718)
Supplement: Supplementary file 1 [file Data_Sheet_1.PDF]

Rapid review process summary table

Review element	Standard realist review	Adaptation in this review	Justification
Timeframe	Often multi-year, iterative across several cycles	Designed to be completed within eighteen months	Timeliness: tie into ongoing work in the field and policy relevance
Stakeholder involvement	Stakeholders engaged iteratively throughout review and theory refinement	Stakeholders consulted at key points only (initial question formation, sense-checking final findings)	Policy-relevance: Maintain practice and policy relevance while ensuring input is feasible, also for stakeholders
Conventional search strategy	Exhaustive and comprehensive	Focused on 6 major databases, limited to 2014-2024	Feasibility: Pragmatic scope to maintain speed while screening, time limit set after comparison to results from 2004-2024 as fewer interventions listed for 2004-2014. Citation chasing for all included to TiAb screening ensured relevant articles from 2004-2014 could still be included.
Tailored search strategy	Extensive, multi-modal and iterative	Supplementary searching used but purposively and stopped when saturation reached	Feasibility: Transparent stopping rules were applied to balance urgency and feasibility e.g. further handsearching for full texts associated with conference abstracts to see if these could contain adequate primary data, but not contacting all authors to request further primary data
Screening	Typically, dual screening at all stages	Calibration with dual screening on 15% sample; remainder screened by one reviewer with conflict resolution procedure	Feasibility: Ensures consistency while prioritizing speed at initial TiAb screening stage where decisions were comparatively more straightforward
Data extraction	Full coding by at least two reviewers across all studies	Primary extraction conducted by one reviewer then cross-checked by a second reviewer independently	Timeliness: Ensures consistency and rigour while prioritizing speed and timeliness
Programme theory development	Iterative cycles of theory testing and refinement	Initial deductive mapping of intervention families; refinement of theories with advisory board	Timeliness: Condenses cycles while retaining theory-driven synthesis
Outputs	Full realist theory refinement across several CMOs	Prioritised programme theories, families of interventions, visual models, and policy/practice recommendations	Policy-relevance: Ensures actionable findings within timely manner
