# Supplementary material for: Social prescribing for refugee populations: a protocol for a rapid realist review of international evidence
Source: Front Public Health. 2026 Feb 16;14:1754718. doi: 10.3389/fpubh.2026.1754718 (PMC12950807; doi:10.3389/fpubh.2026.1754718)
Supplement: Supplementary file 2 [file Data_Sheet_2.PDF]

PubMed (incl. Medline)

Refugee terms block: ((refugee*) OR (asylum seeker*) OR (displaced person*) OR ("forced migrant") AND

Social concept terms block: ("social prescribing"[All Fields] OR "social capital"[All Fields] OR "interpersonal relations"[All Fields] OR "social integration"[All Fields] OR "social cohesion"[All Fields] OR "social interaction"[All Fields] OR "social skills"[All Fields] OR "social relationships"[All Fields] OR "social inclusion"[All Fields] OR "social adjustment"[All Fields] OR "community participation"[All Fields] OR "community involvement"[All Fields] OR "community resources"[All Fields] OR "peer support"[All Fields]) AND

Intervention context: ("review"[All Fields] OR "intervention"[All Fields] OR "project"[All Fields] OR "program*"[All Fields] OR "trial"[All Fields] OR "study"[All Fields])

Filters applied: 2014-2024

Date searched: 28.10.2024

Web of Science

Refugee terms block: ((((ALL=(Refugee*)) OR ALL=(Asylum seeker*)) OR ALL=(Displaced person*) OR ALL=("forced migrant")) AND

Social concept terms block: (((((((((((((((ALL=("social prescribing")) OR ALL=("social capital")) OR ALL=("interpersonal relations")) OR ALL=("social integration")) OR ALL=("social cohesion")) OR ALL=("social interaction")) OR ALL=("social skills")) OR ALL=("social relationships")) OR AB=("social inclusion")) OR ALL=("social adjustment")) OR ALL=("community participation")) OR ALL=("community resources")) OR ALL=("peer support")) OR ALL=("mentoring")) OR AB=("bridging")) AND

Intervention context: ((((((ALL=("review")) OR ALL=("intervention")) OR ALL=("project")) OR ALL=("program*")) OR ALL=("trial")) OR ALL=("study"))

Filters applied: 2014-2024

Date searched: 28.10.2024

Embase

Refugee terms block: ('refugee'/exp OR 'refugee' OR 'asylum seeker'/exp OR 'asylum seeker' OR 'forced migrant'/exp OR 'forced migrant') AND

Social concept terms block: ('social prescribing'/exp OR 'social prescribing' OR 'social capital'/exp OR 'social capital' OR 'interpersonal relations'/exp OR 'interpersonal relations' OR 'social integration'/exp OR 'social integration' OR 'social cohesion'/exp OR 'social cohesion' OR 'social interaction'/exp OR 'social interaction' OR 'social skills'/exp OR 'social skills' OR 'social

relationships' OR 'social inclusion'/exp OR 'social inclusion' OR 'social adjustment'/exp OR 'social adjustment' OR 'community participation'/exp OR 'community participation' OR 'community involvement' OR 'community resources'/exp OR 'community resources' OR 'peer support'/exp OR 'peer support' OR 'mentoring'/exp OR 'mentoring' OR 'bridging') AND

Intervention context: ('review'/exp OR 'review' OR 'intervention'/exp OR 'intervention' OR 'project'/exp OR 'project' OR 'program'/exp OR 'program' OR 'trial'/exp OR 'trial' OR 'study'/exp OR 'study')

Filters applied: 2014-2024

Date searched: 28.10.2024

Psycinfo

Refugee terms block: (TX "refugee" OR TX "asylum seeker" OR TX "displaced person" OR TX "forced migrant") AND

Social concept terms block: (TX "social prescribing" OR TX "social capital" OR "interpersonal relations" OR TX "social integration" OR TX "social cohesion" OR TX "social interaction" OR TX "social skills" OR TX "social relationships" OR TX "social inclusion" OR "social facilitation" OR TX "social adjustment" OR TX "community involvement" OR TX "community resources" OR TX "acculturation" OR TX "mentoring" OR TX "peer support") AND

Intervention context: (TX "review" OR TX "intervention" OR TX "project" OR TX "program*" OR TX "trial" OR TX "study")

Filters applied: 2014-2024

Date searched: 28.10.2024

CINAHL

Refugee terms block: (TX "refugee" OR TX "asylum seeker" OR TX "displaced person" OR TX "forced migrant") AND

Social concept terms block: ("social prescribing" OR "social capital" OR "interpersonal relations" OR "social integration" OR "social cohesion" OR "social interaction" OR "social skills" OR "social relationships" OR "social inclusion" OR "social facilitation" OR "social adjustment" OR "community involvement" OR "community resources" OR TX "acculturation" OR TX "mentoring" OR "peer support") AND

Intervention context: (TX "review" OR TX "intervention" OR TX "project" OR TX "program*" OR TX "trial" OR TX "study")

Filters applied: 2014-2024

Date searched: 28.10.2024

SCOPUS

Refugee terms block: (TITLE-ABS-KEY("refugee") OR TITLE-ABS-KEY("asylum seeker") OR TITLE-ABS-KEY("displaced person") OR TITLE-ABS-KEY("forced migrant")) AND

Social concept terms block: (TITLE-ABS-KEY("social prescribing") OR TITLE-ABS-KEY("social capital") OR TITLE-ABS-KEY("interpersonal relations") OR TITLE-ABS-KEY("social integration") OR TITLE-ABS-KEY("social cohesion") OR TITLE-ABS-KEY("social interaction") OR TITLE-ABS-KEY("social skills") OR TITLE-ABS-KEY("social relationships") OR TITLE-ABS-KEY("social inclusion") OR TITLE-ABS-KEY("social facilitation") OR TITLE-ABS-KEY("social adjustment") OR TITLE-ABS-KEY("community involvement") OR TITLE-ABS-KEY("community resources") OR TITLE-ABS-KEY("acculturation") OR TITLE-ABS-KEY("mentoring") OR TITLE-ABS-KEY("peer support")) AND

Intervention context: (TITLE-ABS-KEY("review") OR TITLE-ABS-KEY("intervention") OR TITLE-ABS-KEY("project") OR ALL("program*") OR TITLE-ABS-KEY("trial") OR TITLE-ABS-KEY("study"))

Filters applied: 2014-2024

Date searched: 28.10.2024
